# 142 Mountaineering – an opportunity for inclusion

**DOI:** 10.1093/eurpub/ckae114.017

**Published:** 2024-09-26

**Authors:** Suzana Pustivšek, Marjeta Čič, Jurček Nowakk, Maja Filipčič

**Affiliations:** National Institute Of Public Health Of Slovenia, Ljubljana, Slovenia; Alpine association of Slovenia, Preddvor, Slovenia; Alpine association of Slovenia, Preddvor, Slovenia; Faculty of education, Ljubljana, Slovenia

## Abstract

**Purpose:**

Mountaineering play an important role in Slovenian society. In recent years, we have seen an attempt to include people with disabilities into Mountaineering. They have fewer opportunities to participate in recreational activities in an accessible way. The aim of the project is to increase the health-promoting physical activity of people with disabilities/special needs and to improve social integration through hiking. It also aims to improve inclusion by creating a supportive and safe environment for all participants. The project will develop and test methods for inclusion of different groups that can be implemented on a broad scale.

**Project:**

Part of Alpine association of Slovenia is also a mountaineering committee for people with disabilities/special needs. The committee is active in two main areas: firstly, in training experts (guides, teachers, social workers, experts in other disability organisations) and volunteers with the basic skills to conduct mountaineering activities for the target group and secondly, in organising free mountain tours for people with disabilities to improve their everyday life, give them equal opportunities to experience nature, help them socialise and motivate them to lead a more active lifestyle. Several stakeholders are involved in the organisation and implementation of the activities: Sonček – Cerebral Palsy Association of Slovenia, Slovenian Paraplegic Association, Dystrophy Society of Slovenia, Multiple Sclerosis Association of Slovenia and Slovenian Scouts. The evaluation of the project is based on the number of training programmes and walks carried out per year, the number of participants and their subjective evaluation of the individual walks. in 2023, 39 events (2632 participants) were organised and 4 repetitions (78 participants) of the training programme were carried out.

**Conclusion:**

The project is a unique platform for promoting integration in sport and health-enhancing physical activity, which has a strong impact on the biological, sociological, and psychological aspects of participants’ health. The project is an example of good practise of inclusion in HEPA.

